# Status of the use and compliance with malaria rapid diagnostic tests in formal private health facilities in Nigeria

**DOI:** 10.1186/s12936-015-1064-x

**Published:** 2016-01-04

**Authors:** Olugbenga A. Mokuolu, Godwin N. Ntadom, Olufemi O. Ajumobi, Roberts A. Alero, Robinson D. Wammanda, Olanrewaju T. Adedoyin, Henrietta U. Okafor, Adekunle D. Alabi, Friday A. Odey, Chimere O. Agomo, Kate U. Edozieh, Tolulope O. Fagbemi, Ahmad M. Njidda, Seye Babatunde, Emmanuel C. Agbo, Nnamdi B. Nwaneri, Emmanuel D. Shekarau, Temitope O. Obasa, Nnenna M. Ezeigwe

**Affiliations:** Department of Paediatrics and Child Health, College of Health Sciences, University of Ilorin, Ilorin, Kwara Nigeria; National Malaria Elimination Programme, Federal Ministry of Health, Abuja, Nigeria; University of Lagos, Lagos, Nigeria; Ahmadu Bello University, Zaria, Nigeria; University of Nigeria, Enugu, Nigeria; Olabisi Onabanjo University, Sagamu, Nigeria; University of Calabar, Calabar, Nigeria; Nigeria Institute of Medical Research, Lagos, Nigeria; Foundation for Charity and Community Health Nigeria, Abuja, Nigeria; World Health Organization, Abuja, Nigeria

**Keywords:** Malaria rapid diagnostic tests, Formal private health facility, Compliance, Nigeria

## Abstract

**Background:**

Nigeria has the largest number of malaria-related deaths, accounting for a third of global malaria deaths. It is important that the country attains universal coverage of key malaria interventions, one of which is the policy of universal testing before treatment, which the country has recently adopted. However, there is a dearth of data on its implementation in formal private health facilities, where close to a third of the population seek health care. This study identified the level of use of malaria rapid diagnostic testing (RDT), compliance with test results and associated challenges in the formal private health facilities in Nigeria.

**Methods:**

A cross-sectional study that involved a multi-stage, random sampling of 240 formal private health facilities from the country’s six geo-political zones was conducted from July to August 2014. Data were collected using health facility records, healthcare workers’ interviews and an exit survey of febrile patients seen at the facilities, in order to determine fever prevalence, level of testing of febrile patience, compliance with test results, and health workers’ perceptions to RDT use.

**Results:**

Data from the 201 health facilities analysed indicated a fever prevalence of 38.5 % (112,521/292,430). Of the 2077 exit interviews for febrile patients, malaria testing was ordered in 73.8 % (95 % CI 71.7–75.7 %). Among the 1270 tested, 61.8 % (719/1270) were tested with microscopy and 38.2 % (445/1270) with RDT. Compliance to malaria test result [administering arteminisin-based combination therapy (ACT) to positive patients and withholding ACT from negative patients] was 80.9 % (95 % CI 78.7–83 %). Compliance was not influenced by the age of patients or type of malaria test. The health facilities have various cadres of the health workers knowledgeable on RDT with 70 % knowing the meaning, while 84.5 % knew what it assesses. However, there was clearly a preference for microscopy as only 20 % reported performing only RDT.

**Conclusion:**

In formal private health facilities in Nigeria there is a high rate of malaria testing for febrile patients, high level of compliance with test results but relatively low level of RDT utilization. This calls for improved engagement of the formal private health sector with a view to achieving universal coverage targets on malaria testing.

## Background

Malaria remains a major public health problem with over 3.2 billion of the world’s population at risk, of which 1.2 billion people are at high risk [1, 2]. Over the last 15 years there has been concerted effort through global partnerships to mobilize resources for the deployment of cost-effective tools towards a significant reduction in malaria morbidity and mortality, with a decrease in malaria incidence by 30 % globally, and by 34 % in Africa [2–5]. Furthermore, the prevalence of malaria parasite infection (including both symptomatic and asymptomatic infections) fell from 173 million in 2000 to 128 million in 2013, a reduction of 26 % in Africa [2]. These reductions are largely due to increased deployment of long-lasting, insecticide-treated nets (LLINs), increased availability of highly effective artemisinin-based combination treatment (ACT) and improved diagnosis of malaria. While deployment of LLINs and ACT have been significantly scaled up towards universal coverage levels in most countries, scale up of malaria diagnosis has been much slower, due to its greater dependence on a number of systemic issues influencing availability and usage in guiding anti-malarial prescriptions.

In 2010 the WHO recommended universal testing before treatment in suspected cases of malaria [6, 7]. Implementing this requires expanded access to malaria diagnosis at all levels and sectors of health care delivery. The options for expanding access are scaling up microscopy and the use of malaria rapid diagnostic tests (RDTs). Malaria microscopy has inherent challenges as a primary tool for scaling up diagnosis. These challenges include quality control issues, supportive infrastructure, cost and manpower capacity needs [8–13]. The RDTs on the other hand, while not a substitute for microscopy, possess qualities that address many of the limitations of microscopy. This makes the use of RDT a more feasible strategy for expanding access to diagnosis [14–18]. Although, use of RDT has its own challenges e.g. use of excess buffer/inappropriate sample well, storage-related challenges but they are easily overcome with training and routine supervision. Several recent studies have continued to validate the usefulness and reliability of RDTs for malaria diagnosis in both children and adults [19–24]. The acceptability of RDT among care-seekers attending public healthcare facilities, patent medicine vendors and private health retailers has also been documented by research [25–32]. In addition, use of RDTs for malaria diagnosis have been reported to be cost-effective in the treatment of malaria, especially in relation to the cost of ACT and in the face of declining prevalence of malaria [11, 33–37].

Consistent with WHO recommendation, Nigeria, in 2011, updated the National Malaria Treatment Guidelines to reflect universal testing before treatment for suspected cases of malaria [29]. Prior to this, the policy in Nigeria recommended testing before treatment in children older than 5 years and in adults, with a more liberal bent for presumptive or syndromic treatment in children under 5 years. One of the objectives of the National Malaria Strategic Plan 2014–2020 is to test all care-seeking persons with suspected malaria using RDT or microscopy by 2020 [30]. Attaining this objective makes it imperative for all levels and segments of the Nigerian health system to have access to, and appropriately utilize, malaria diagnostic tools. To this end the national malaria elimination programme (NMEP) commissioned implementation research to guide RDT scale-up and deployment to primary healthcare facilities in the public sector [38].

The private health sector in Nigeria is very large, consisting of formal tertiary, secondary and primary health facilities, pharmacies and informal drug retailers. The National Health Directory listed 34,173 health facilities in Nigeria at December 2011 [39]. This has been updated recently to 34,423 health facilities in the country [40]. Forty per cent of these registered health facilities belong to the private sector, especially at primary and secondary health care level [39]. Regarding health-seeking behaviour, about 60 % of people seek treatment for fever from private health facilities while 40 % go to the public sector [41]. Key implementing partners involved with malaria case management (including training on microscopy and RDT use) in the private health sector in Nigeria include the society for family health (SFH), DfID funded support for national malaria programme (SuNMaP) and PMI-funded malaria action programme for states (MAPS). The UNITAID is currently working on RDT roll-out to the private sector.

Studies have indicated the inherent differences in healthcare practices across the different health sectors (private vs public) and even within the same sector [42–45]. Considering that the formal private health facilities are a major contributor to healthcare delivery in Nigeria, their compliance with national policies and treatment guidelines is critical to attainment of the overall goal of the NMEP. The independence by clients in dictating their service needs and overall mode of engagement of the formal private health sector, that is profit driven, precludes any extrapolation of data from the public sector to the formal private health sector.

In light of the dearth of knowledge on the status of RDT use in the formal private health sector in Nigeria, this study was undertaken to identify practices related to the use of RDT in private health facilities. With lessons learnt from the surveys in the public health sector and informal private sector, it was expected that the findings from this study on formal private health facilities would complete the loop of understanding needed to guide continued deployment and use of RDTs in the country. Improved diagnosis will contribute to better treatment of malaria, thereby contributing further to reduction in malaria mortality and better tracking of progress made.

## Methods

This was a cross-sectional study to assess RDT use and its associated challenges in formal private health facilities in Nigeria. Formal private health facilities were defined as hospitals, clinics or maternity homes registered by the state ministry of health (SMOH) as a private health facility offering orthodox medical care. This excludes health facilities registered primarily as pharmacy/patent and proprietary medicine vendors and/or for laboratory services. State Hospital Management Boards register, regulate and license private health facilities. For the purpose of this study, only private hospitals were enrolled.

### Study sites

Nigeria is a West African country with 36 states and 774 Local Government Areas (LGAs) and Federal Capital Territory (FCT). For administrative convenience and representation, the country uses geo-political zoning, comprising northcentral (six states and the FCT); northeast (six states); northwest (six states); southeast (five states); southsouth (six states and southwest (six states). Malaria transmission has been intense, stable and holo-endemic in most parts of the country. Recent reports suggest some changes over time, such that as at 2010, 85 % of Nigerians lived in areas supporting meso-endemic transmission, 15 % lived under conditions of hyper-holo-endemicity, with some areas within FCT and northeastern states supporting hypo-endemicity [46]. Seasonality, intensity and duration of malaria transmission in Nigeria varies according to five ecological strata. These include mangrove swamps, rain forest, guinea-savannah, sudan-savannah and sahel-savannah. The duration of the season decreases from the south to the north, being perennial in duration in most of the south but lasting 3 months or less in the northeastern region bordering Chad [47]. In the north, high transmission season is between July and November and in the south is between April and October with bimodal peaks in July and September.

### Study setting

The study was designed to determine the prevalence of fever, the practices related to RDT use, and to understand the perception of a sample of health workers to RDT. It was anticipated that there could be significant variations in the quality of health facility records, thereby limiting their aggregation to answer the research questions. Three sets of tools were designed to address the study objectives:

Health facility records: this was to focus on the record of illness episodes as documented in order to establish the proportion of complaints that were fever. The facility interview focused on attendance rate and proportion of fever cases over the preceding 6 months of the study. Information on the facility stock level, utilization of malaria commodities, supply chain management system, state of infrastructure and challenges encountered was also captured in the questionnaires. The records were extracted from National Health Management Information registers provided with support from the Global Fund through SFH or any other hospital register where these were not available.

Healthcare workers’ interviews conducted to appraise the practices of the health facilities as reported by the health workers. Data were collected from the officers-in-charge or health personnel that dispense diagnostics.

Exit interview of febrile patients: aimed at systematically and prospectively collating perceptions of patients regarding RDT at the private health facilities. In addition, it was expected to provide a reliable denominator to facilitate the estimates related to level of diagnosis and compliance with test results.

### Sampling technique

**Fig. 1:**
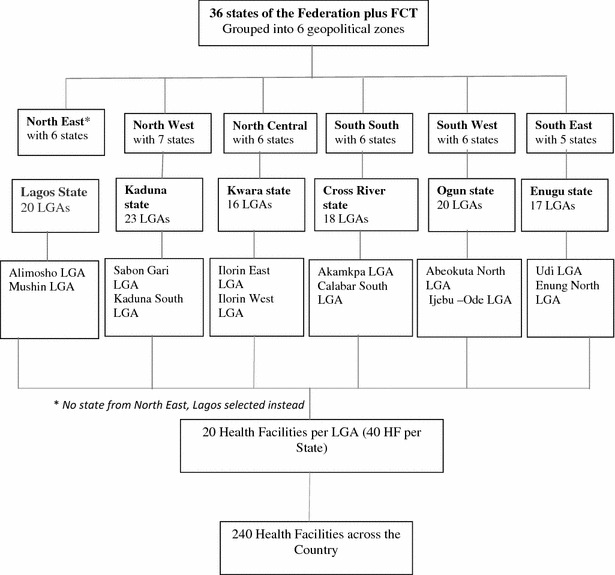
Flow chart on sampling procedure

The health facilities were selected using a multi-staged, random sampling technique. In the first stage, six states were randomly selected using geo-political zones as sampling frames viz; Cross-River in mangrove ecological zone, Enugu and Lagos in rain forest, Kaduna in sudan-savannah), Kwara and Ogun in guinea-savannah. States in the northeast geo-political zone were excluded on account of security reasons, and Lagos State was selected because of its coastal location and status as emerging mega city. In each selected state, two LGAs (one urban and one rural) were randomly selected. From each of the selected LGAs, the health facilities were selected by simple random sampling from a sampling frame of the list of registered private health facilities obtained from the SMOH. Twenty private health facilities were selected from each LGA. In cases where there were less than 20 private health facilities available in a selected LGA, data were collected from health facilities in a contiguous LGA to complete the sample size. Thus, data were collected from a total of 240 private health facilities across the country (Fig. 1). One health facility survey questionnaire, one health worker interview questionnaire and ten exit interviews per facility was administered across health facilities surveyed.

### Study procedure

Preliminary activities consisted of meetings between the Principal Investigator (PI), State team leaders and other key members of the study team to fine-tune the data capture tools and data analysis methods. Data collection tools comprised questionnaires to capture previous medical records, to ascertain hospital attendance and proportion of febrile patients tested and treated appropriately, and exit interview questionnaires to validate reported practice and community perception of RDT. The questionnaires were pre-tested in health facilities in the Federal capital city where the study would not be carried out and observations made during the pilot informed revision of the final data capture tools.

At each study site, the study team comprised the team leader, six interviewers, the state epidemiologist, state malaria programme officer and malaria focal persons at the two LGAs. Field statisticians were assigned, one each, by NMEP to various study sites. A two-day training workshop was organized at each site prior to commencement of field work. During the workshops, the state team members were familiarized with the objectives of the study and introduced to the data capture tools. Ethical clearance for the study was obtained from the National Health Research and Ethics Committee (NHREC).

### Data collection

Data were collected using study instruments over 22 days in each state from July to August 2014. Data collected were reviewed and cleaned each day by the state team leaders and field statisticians, who subsequently entered data in computer. At the end of data collection in each state, the field statisticians sent their data set to the central study statistician for pooled entry.

### Data management

Data entry was done using EPI info version 3.5.4. There was onsite data entry by each of the team data management officers. Thereafter, data from the states were merged to create a common database. Simple frequencies were generated to facilitate the process of data cleaning. Queries were generated and clarifications sought from the respective teams as applicable. The cleaned dataset was subsequently exported to SPSS version 22 for analysis.

Frequency tables and charts were used to present simple proportions with 95 % confidence intervals (CI) using the online Vassarstat^®^ application. Comparison of categorical variables and specific outcome was carried out using the Chi square test, while the comparison between continuous variables and specific discrete outcomes was done by Student *t* test. For all the statistical analysis, the level of significance was set at p < 0.05, 95 % CI.

### Limitation

This study did not address quality assurance of malaria diagnostics in the private sector. Record keeping was generally poor precluding a comparison of the data extracted from the registers among the study sites. The decision to use mainly the exit survey in defining denominators for the various measurements was informed by the anticipation of this possibility.

## Results

**Fig. 2:**
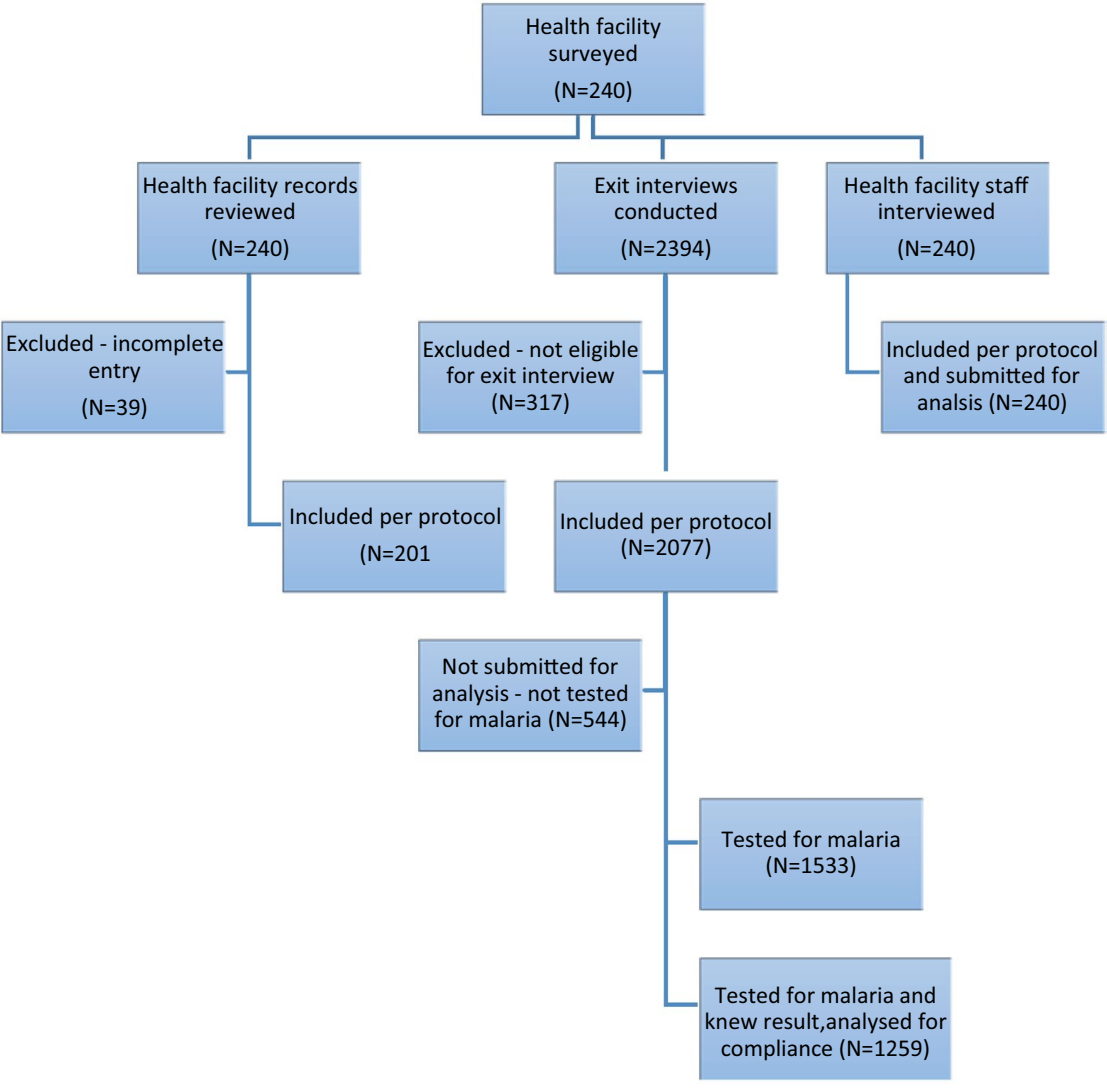
Description of algorithm for data collection and enrolment of subjects

The study was conducted using three instruments: summary of medical records, exit interviews and key health staff interviews. The flow of data collection is summarized in Fig. 2. Of the 232 facilities with health workers’ responses to malaria testing practices, microscopy was done in 98 (42.2 %) health facilities, RDT in 46 (19.8 %), both RDT and microscopy in 74 (31.8 %), while 16 (6.8 %) reported that they do not carry out any malaria test in the facility. Of the 240 health facilities, 107 (44.6 %) currently had RDT in stock though 110 (45.8 %) use RDT routinely.

### Prevalence of fever from health facilities

Data of hospital registers from 240 healthcare facilities were assessed to determine prevalence of fever among hospital consultations; 39 incomplete records (Kwara: 27, Kaduna: 7, Cross-River: 3 and Ogun: 2) were excluded; data were analysed for 201 health facilities (Fig. 2). The prevalence of fever in relation to total attendance at the health facilities was 38.5 % (112,521/292,430) within the period of 6 months (January–June 2014) (Table 1).

**Table 1:** Record of attendance in sampled formal private health facilities in Nigeria

State (no of HF)^a^	Total attendance	Total fever	Percentage with fever
Cross River state (n = 37)	17,745	8374	47
Enugu (n = 40)	73,670	29,924	41
Kaduna (n = 33)	65,879	29,317	45
Kwara (n = 13)	39,292	11,078	28
Lagos (n = 39)	47,506	16,904	36
Ogun (n = 38)	48,338	16,924	35
Total (n = 200)	*292,430*	*112,521*	*38.5*

^a^Data was incomplete in some states

### Health facility practices from exit interviews

Concomitant symptoms: A total of 2077 exit interviews were conducted for febrile patients across the study sites (Fig. 2). In addition to fever, generalized weakness (60.9 %), cough/catarrh (44.5 %), vomiting/diarrhoea (39.9 %), pallor (8.1 %) and convulsion (1.3 %) were reported as presenting symptoms (Table 2). The distribution of these symptoms was similar across the sites (Table 2).

**Table 2:** Frequency of complaints among 2077 respondents with fever by study sites

State	Fever	Concomitant symptoms				
		General weakness	Cough catarrh	Diarrhoea vomiting	Convulsions	Pallor
C/River	344	258	210	120	10	3
Enugu	385	181	223	154	7	19
Kaduna	398	211	140	170	0	19
Kwara	375	245	152	170	6	18
Lagos	327	268	149	134	1	49
Ogun	248	101	109	132	4	71
Total	*2077*	*1264 (60.9)*	*983 (44.5)*	*880 (39.9)*	*28 (1.3)*	*179 (8.1)*

Multiple symptoms

Use of malaria diagnostics: Out of the 2077 febrile patients, 1533 had their blood tested during the hospital visit (Fig. 2), giving a testing rate of 73.8 % (95 % CI 71.7–75.7 %) for febrile patients visiting the formal private health facilities. Out of the 1533 patients who had their blood tested, 1259 (82.1 %) were told the outcome of the test carried out. Among those tested 1164 (75.9 %) knew the type of test carried out (microscopy or RDT). Of those that knew the test type, 719/1164 (61.8 %) were tested with microscopy, while 445 (38.2 %) were tested with RDT. Of the patients who knew the outcome of their tests, 1079 (85.7 %) were positive for malaria while 180 (14.3 %) were negative. Among respondents who provided information on payment for these laboratory services, 90.1 % of them reported that they paid for their blood test out of which 53.7 % knew the amount paid.

Reported compliance with test results: Compliance was computed from the total of those positive for malaria that had ACT and those negative for malaria that did not receive ACT/anti-malarial expressed as a proportion of the total tested. Findings in this regard showed that of 1078 patients whose malaria test results were reported positive, 892 (82.7 %) received ACT while out of 181 patients whose malaria tests were reported as negative, 126 (69.6 %) did not receive ACT. Thus, ‘compliance’ to malaria test result in administering ACT was 80.9 % (95 % CI 78.7–83.0 %). The compliance was 75 % in Enugu and consistently above 80 % in all the other states with Kwara having the highest at 87 % (Table 3). Stratifying the reported compliance practices by the type of test performed and by age (<5 vs 5 years and above) indicated that there were no apparent differences in the compliance patterns on the basis of these parameters (Table 4).

**Table 3:** Compliance with use of ACTs

States	Test Result Known	Positive + ACT	Negative–ACT	Compliance (%)
C/River	262	191	19	80.2
Enugu	203	134	18	74.9
Kaduna	293	214	34	84.6
Kwara	177	143	11	87.0
Lagos	187	118	27	77.5
Ogun	138	92	17	79.0
Total	*1259*	*892*	*126*	*80.9*

**Table 4:** Malaria Test result and ACT use

Selection	n	ACT given		Chi square P-value	Compliance (95 % CI)
		Yes (%)	No (%)		
All tests	1259			<0.0001	80.9 (78.7–83.0)
Positive		892 (82.7)	186 (17.3)		
Negative		55 (30.4)	126 (69.6)		
Microscopy only	633			<0.0001	83.1 (80.0–85.8)
Positive		484 (85.7)	81 (14.3)		
Negative		26 (38.2 %)	42 (61.8 %)		
mRDT only	412			<0.0001	85.7 (82.0–88.7)
Positive		291 (88.7)	37 (11.3)		
Negative		22 (26.2)	62 (73.8)		
Under 5	117			<0.0001	79.5 (71.3–85.8)
Positive		84 (79.2)	22 (20.8)		
Negative		2 (18.2)	9 (81.8)		
5-years & above	1081			<0.0001	80.9 (78.4–83.1)
Positive		760 (82.8)	158 (17.2)		
Negative		49 (30.1)	114 (89.9)		

### Health facility practices from health workers’ in-depth interviews

Two-hundred and forty health workers were interviewed in order to triangulate their information with what was obtained in the exit interviews so as to understand practices within the health facilities in relation to malaria diagnosis and treatment. The distribution of respondents shows that the majority were doctors 69 (29.1 %), followed by nurses 51 (21.5 %), medical laboratory scientists 50 (21.1 %), auxiliary nurses 31 (13.1 %), community health extension workers 26 (11.0 %), community health workers 7 (3.0 %) while pharmacists, 3 (1.3 %), were least represented.

Health facilities with knowledgeable health workers: Among the 240 health workers, 70.0 % knew the meaning of RDT, while 84.5 % knew what RDT assesses. Of the 229 that responded to the question on specimen used for RDT, 210 (91.7 %) knew that blood was used for the test, one (0.4 %) said urine was used for the test while 18 (7.9 %) did not know the specimen used for the test. Of the 223 respondents to the question on how to carry out RDTs, 160 (71.7 %) knew how to carry out RDT while 63 (28.3 %) did not know. Practices of health workers were equally assessed. In 87.8 % (95 % CI 82–92 %) of health workers, results of RDT were available at the time of treating patient. The result of RDT was used in 73.7 % (95 % CI 67–79 %) to determine treatment. Reported mean time for performing RDT was 11.9 [standard deviation (SD) 7.7] minutes. The mean amount paid for the test was N375 (SD 175), an equivalent of $2.5 as at the time of the study. Perception-wise: 75 % (95 % CI 73–85 %) of health workers were of the opinion that RDT was useful in their facilities. In addition, the majority reported it took a short time, was easy to conduct, and that it did not interfere with clinic activities (Table 5).

**Table 5:** Perceptions and practices from the key health workers’ interviews on malaria rapid diagnostic use in the health facilities

Item assessed	n	Responses	Percent (95 % CI)
Health facilities with knowledgeable health workers		*Correct knowledge*	
Meaning of RDT	231	168	72.7 (67–78)
What RDT assesses	232	196	81.7 (79–89)
Specimen required	229	210	91.7 (87–95)
Report ability to do test	223	160	71.7 (66–77)
Health facility mRDT practices by health workers		*Practice is yes*	
Dedicated health worker for mRDT	184	125	67.9 (61–74)
Use mRDT to determine treatment	186	137	73.7 (67–79)
Use mRDT routinely	185	107	107 (52–66)
Result available when treating patient	156	137	87.8 (82–92)
Perception in the health facilities on the use of mRDT Variable		*Favorable perception*	
Useful for malaria diagnosis	166	132	79.5 (73–85)
Takes short time	172	155	90.1 (85–94)
Easy to conduct	131	126	96.2 (91–98)
Does not interfere with clinic activities	129	122	94.6 (89–97)

## Discussion

Fever prevalence from the health facilities’ records was 38.5 %. The prevalence varied from 28 to 47 % across the various geo-political zones of the country. The fever prevalence in this study is lower than previously reported rate of 60 % among outpatients [29]. It is also slightly lower than the weighted average of 43 and 45 % reported among children taken to formal hospitals in northeastern and southern parts of the country, respectively [48]. The reasons for this lowering in fever prevalence may be connected to overall reductions and gains made in the control of malaria and acute respiratory tract infections [2, 46]. However, an additional possibility is that there is a wider range of non-febrile conditions that may be seen at the formal private health facilities since most users of such facilities tend to subscribe as families and they report there as their primary point of call for a variety of non-febrile conditions.

Among the 2077 patients with fever, there were a number of concomitant symptoms. The most frequent being generalized weakness, cough, vomiting, and diarrhoea. The predictive importance of the individual symptoms in relation to malaria was not undertaken in this study. However, the combination of these features continues to underscore the challenge of developing a reliable clinical algorithm for the diagnosis of malaria. Most efforts in this regard have resulted in relatively poor sensitivity and specificity, making the diagnosis of malaria solely reliant on parasitological testing [49–51]. This was the basis of Nigeria’s recent policy change, in alignment with the global recommendation on testing before treatment, that until proven by test, a case of fever regardless of the clinical symptomatology, is ‘suspected’ malaria [52]. This study’s finding on the status of the implementation of the ‘test before treatment’ strategy in a national survey of private health facilities is therefore instructive.

This study showed that about three-quarters, 73.8 % (95 % CI 71.9–75.7 %) of fever cases were tested for malaria in private health facilities in Nigeria. This is much higher than testing rates reported in public health facilities in the country [38]. The 2013 Nigeria Demographic and Health Survey (NDHS) reported that only 11.1 % of children under 5 years with fever had blood taken for testing [41]. The finding could be considered counter-intuitive when it is considered that unlike public facilities, private facilities, which are classified under ‘alternative healthcare system’ in Nigeria’s three-tier structure (National Health Policy), are less supervised for quality of care and adherence to guidelines. Conversely, this finding is comforting, given the fact that more Nigerians seek care for fever in private health facilities despite the relatively larger size of the public health sector [39, 41]. It perhaps lends some support to the underrated successes of the country’s decade-old Roll Back Malaria campaign aimed at achieving universal coverage of malaria interventions. It also probably reflects the support from RBM partners and the Global Fund to malaria case management in the private sector.

A further possible reason for the high testing rate in private health facilities could be motivation for income generation, fear of litigation or simply an adherence to policy/practice regulation. Anecdotal evidence in informal medical discourse in Nigeria favours the notion that, because of economic gain, private practitioners are ‘quick on the draw’ to order tests for care-seekers. That said, the outcome of this study regarding the status of testing for malaria before treatment in private health facilities is commendable.

Testing with RDTs was not observed to be as common as testing by microscopy, which, although considered the gold standard, requires more stringent conditions [14, 53, 54]. This appears consistent with some other studies where it has been observed that availability of RDTs was limited in a sample of private facilities that were studied [55]. This is probably because formal private facilities are expected to be less resource-constrained than public facilities, even in low- and middle-income countries such as Nigeria, owing to their ‘for-profit’ orientation. There may also be concerns that the quality of RDTs could limit their preference in the private health facilities [55]. Concerns about quality may have been why the double test of RDT and microscopy, was performed. The recent scale-up of RDT deployment in the NMEP has been largely directed at the public sector. Among the febrile patients, there was a very high prevalence of malaria positivity. This would appear somewhat discordant with the overall reduction in fever prevalence as observed in hospitals. Considering that microscopy was the dominant means of diagnosis, this finding raises concern about its quality assurance. This observation may need further exploration in studies that will focus significantly on quality assurance issues for malaria diagnosis in private health facilities. In the meanwhile, there might be the need for NMEP and RBM partners to strengthen quality assurance in malaria diagnosis in private sector by ensuring compliance with the provision of implementation guide for parasite-based diagnosis of malaria in Nigeria. Current findings will however suggest the need for more concerted outreach to formal private health facilities in order to improve their uptake of RDTs, especially given the challenges on quality assurance with malaria microscopy.

The high rate of test-before-treatment practice in the private facilities was also complimented by a high level of compliance to the result of the malaria test. This study’s composite measure of ‘compliance’ to appropriate treatment with ACT following malaria test result was a high 80.9 % (95 % CI 78.7–83.0 %), inclusive of those whose tests were negative and were not given ACT. The level of compliance was not affected by type of test or age of patients. There is virtually no parallel national study to compare this important finding with, except the NDHS household-based surveys reporting that 6.0 % of children under five with fever were appropriately given ACT, irrespective of test-result status or the health provider type [41]. ‘Compliance’ may also have been incentivised by the prospect of additional economic gain by prescribing a treatment, for a private establishment. However, as noted, the weighting of ‘compliance’ in this study also included not treating with ACT when the malaria test was negative. This balanced rational drug use is one of the cardinal indicators or measures of quality care. Several studies have already reported the cost-effectiveness of the test-before-treatment policy in case management of malaria [33, 35–37, 56]. In this study, the cost of test was about $2.5 and this did not appear to be a disincentive to the testing rate. Overall, 9.3 % of the clients had any form of medical insurance. It is, therefore, unlikely that insurance coverage will be an important explanation for the testing rate in spite of the cost of testing. A peculiarity within the Nigerian system is the general belief that laboratory testing is part of the evidence of the quality of care and may serve as a facilitator of willingness to pay. The authors postulate that this may the underlying factor behind the sustained high testing rate despite the cost of testing.

The spread of cadre of health workers reported in the present study is similar to that reported by Uzochukwu et al. [42]. Their study showed that awareness and knowledge by health workers was higher among the public (72.2 %) than the private (50.0 %) health workers. The correct knowledge by over 70 % (range 71.7–91.7) of private health workers reported in the present study was higher than the 61.1 % reported by Uzochukwu et al. [42]. The reason for this could be attributed to training on RDT conducted by several non-governmental organizations working in collaboration with the NMEP. These workers in the private health facilities reported a higher level of 79.5 % in the usefulness of RDT for malaria diagnosis than the 65.6 % health workers reported by the previous study [38].

The perception of the usefulness of RDT for malaria diagnosis and treatment among health workers in the private facilities was favourable. The perceived usefulness of the RDT test for diagnosis by the health workers in this study was 79.5 % (95 % CI 73–85 %) against 65.6 % previously reported [42]. Health workers’ opinion that RDT took a “short time” was consistently high with that reported by same authors [38]. These positive attitudes provide important opportunity for the scale up of RDT use in formal private health facilities with information targeted on addressing other areas of concerns that might limit their absolute confidence in the use of RDTs.

Regardless of the point of care, when health providers adhere to or comply with malaria test results, it is expected that there will be a lot of benefit to the health systems, the patients and the health facilities.

## Conclusions

This study revealed an unprecedented report of the status of malaria testing rate, and compliance with malaria test results in private health facilities in Nigeria. In formal private health facilities in Nigeria, there is a high rate of malaria testing for febrile patients, high level of compliance with test results but relatively low level of RDT utilization. This calls for improved engagement of the formal private health sector with a view to achieving universal coverage targets on malaria testing.
